# Food environments select microorganisms based on selfish energetic behavior

**DOI:** 10.3389/fmicb.2013.00348

**Published:** 2013-11-22

**Authors:** Diego Mora, Stefania Arioli, Concetta Compagno

**Affiliations:** Department of Food, Environmental, and Nutritional Sciences, University of MilanMilan, Italy

**Keywords:** lactic acid bacteria, yeast, metabolism, evolution, food microbiology

## Abstract

Nutrient richness, and specifically the abundance of mono- and disaccharides that characterize several food matrixes, such as milk and grape juice, has allowed the speciation of lactic acid bacteria and yeasts with a high fermentation capacity instead of energetically favorable respiratory metabolism. In these environmental contexts, rapid sugar consumption and lactic acid or ethanol production, accumulation, and tolerance, together with the ability to propagate in the absence of oxygen, are several of the “winning” traits that have apparently evolved and become specialized to perfection in these fermenting microorganisms. Here, we summarize and discuss the evolutionary context that has driven energetic metabolism in food-associated microorganisms, using the dairy species *Lactococcus lactis* and *Streptococcus thermophilus* among prokaryotes and the bakers’ yeast *Saccharomyces cerevisiae* among eukaryotes as model organisms.

## INTRODUCTION

What is the role of environmental constraints in streamlining and shaping the energetic metabolism of food-adapted microorganisms? Here, we compare prokaryotic lactic acid bacteria (LAB) and the eukaryote *Saccharomyces cerevisiae* to draw bioenergetic analogies between these two classes of microorganisms that lead us to hypothesize a common evolutionary path. This paper will focus on the evolutionary forces that allow selection between selfish and cooperative energetic behaviors (Pfeiffer et al., 2001) in food-associated microorganisms. The analysis of energetic behaviors will be performed while considering the environmental context in which microorganisms evolve and the environmental changes that occur during microbial growth in a food matrix. Recently, the relevance of selfish and cooperative behaviors to the origins and the evolution of complex microbial communities in natural environments has been discussed (Sachs and Hollowell, 2012). These behaviors demonstrate how metabolic cooperation among microorganisms determines their genomic and metabolic streamlining. Here, we discuss the role of food environments generated by anthropic activities in shaping the energetic metabolism in food-associated microorganisms.

## CHALLENGING METABOLISM WITH ENVIRONMENTAL STIMULI

During growth, every microorganism causes considerable changes in the environmental, chemical, and physical conditions by changing the concentrations of nutrients and organic acids and by other molecules generated through the organism’s catabolism. Certain habitats may fluctuate erratically, whereas others, which are more predictable, may offer the opportunity to prepare in advance for the next environmental change. In this context, microorganisms living in “predictable” fluctuating environments have evolved regulatory machinery to anticipate environmental perturbations by adapting to the perturbations’ temporal order of appearance (Mitchell et al., 2009; Zakrzewska et al., 2011). Food matrixes represent a good example of “predictable” fluctuating environments generated by anthropic activities. Nutrient richness, and specifically, the abundance of mono- and disaccharides that characterize several food matrixes, such as milk and grape juice (in which mono- and disaccharide resources are both large and dense), could have further allowed the speciation and/or domestication of LAB and yeasts with a high fermentation capacity instead of more energetically favorable respiratory metabolism (Bolotin et al., 2004; Fay and Benavides, 2005). *S. cerevisiae* has been exploited for several millennia throughout the world for its ability to produce beer, wine and bread. This widespread use is due to a robust fermentative metabolism. This organism’s ability to degrade sugars into ethanol and CO_2_, even under aerobic conditions, thus using fermentative metabolism instead of respiration, is at the basis of its popularity as “food” yeast and its benefit to human civilization for thousands of years (Piškur et al., 2006).

**FIGURE 1:**
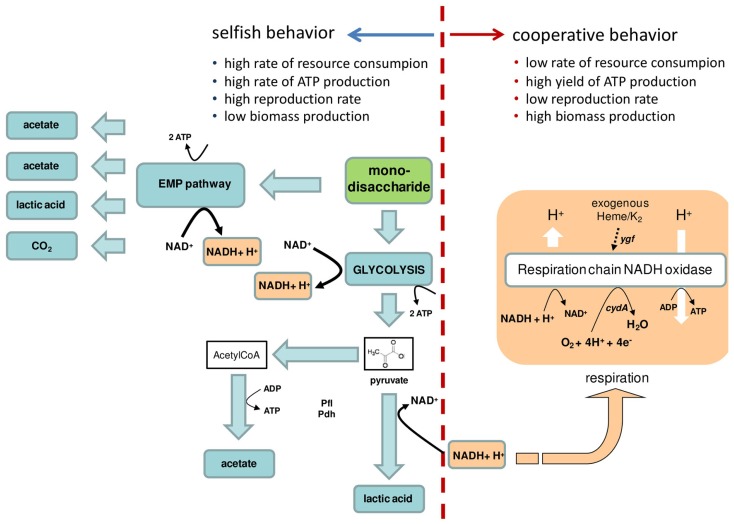
**Simplified representation of “selfish” and “cooperative” energetic behaviors in lactic acid bacteria.** The key characteristics of “selfish” and “cooperative” energetic behaviors are indicated. The dotted red line separates the metabolic reactions that confer a “selfish” or a “cooperative” behavior. In lactic acid bacteria (LAB), mono- or disaccharides are catabolized: (i) to pyruvate via glycolysis, with the production of ATP and NADH, or ii) trough the exose monophosphate pathway (EMP), the heterofermentative pathway. The yellow arrow shows the regeneration of NAD^+^ that occurs during heme-dependent respiration. In *Lactococcus lactis* and in other LAB, the excess pyruvate can be metabolized by pyruvate formate lyase (Pfl), pyruvate dehydrogenase (Pdh) giving a mixed-acid fermentation with production of acetate. *Ygf* is the operon involved in heme homeostasis, and *cydA* is the gene for the cytochrome *bd* oxidase subunit I as described for *L. lactis* (Arioli et al., 2013). Arrows show the metabolic fluxes.

Among bacteria, the seemingly simplistic metabolism of LAB has been exploited throughout history for the preservation of foods and beverages in nearly all societies, dating back to the origins of agriculture (Dunne et al., 2012; Hayden et al., 2013). Certain LAB species have partially lost the genetic information required for respiratory metabolism, resulting in versatile fermentative metabolism. This metabolism involves a homofermentative pathway in which lactic acid is the primary product or a heterofermentative pathway in which lactic acid, CO_2_, acetic acid, and/or ethanol are produced (**Figure 1**). What were the natural habitats that drove the evolution of such peculiar metabolic traits in these microorganisms? The relevant type of environment appeared on Earth at the end of the Cretaceous age (between 145 and 65 mya), when an excess of fruits and therefore increased amounts of fermentable substrates became available to many microbial communities. Additionally, if we consider the availability of a high amount of lactose linked to the appearance of mammals on Earth, the adaptation of certain LAB species to the milk environment should have started in the late Paleocene (between 65 and 23 mya). However, the adaptation of LAB to food fermentations took place between 12000 and 6000 years ago (Dunne et al., 2012; Hayden et al., 2013) a period too short, in evolutionary terms, to justify that human activity directed evolution at the level of energetic metabolism, but sufficiently long to model regulatory mechanisms, and to drive genetic rearrangements through mobile genetic elements and horizontal gene transfer events.

In those environmental contexts, LAB and yeasts could have evolved efficient fermentation metabolisms characterized by a high rate of sugar consumption associated with the accumulation of catabolic products, such as ethanol or lactic acid. It therefore appears that *S. cerevisiae* and LAB shared certain common constraints that have driven the evolution of their energetic metabolisms. What were the evolutionary driving forces that allowed this lifestyle to emerge? The answer depends on two very important parameters: gain and cost. The added value of the rapid utilization of available carbon sources and of the rapid accumulation of fermentation products is that these metabolic traits represent a strategy to efficiently outcompete other microbes in the same environmental context, thus becoming “winning” traits (MacLean and Gudelj, 2006; Piškur et al., 2006). Apparently, both *S. cerevisiae* and certain LAB species have evolved their energetic metabolism to reach maximum fitness in defined environmental niches characterized by high carbohydrate concentration.

## “SELFISH” AND “COOPERATIVE” BEHAVIOR: THE POSSIBILITY OF CHOICE

In contrast to most LAB species, *S. cerevisiae* can use respiro-fermentative metabolism instead of only energetically more favorable respiratory metabolism (2 versus 32 mol of ATP per mol of glucose, respectively, for fermentation and respiration). This phenomenon is called the “Crabtree effect” (Crabtree, 1929). Extensive studies have been performed to study such complexity at the physiological, molecular, and structural levels (Fendt et al., 2010). It has been demonstrated that the Crabtree effect is mainly the result of a wide repression exerted by glucose on respiratory metabolism.

Respiration is characterized by a high yield of ATP production (the number of units of ATP per unit of resource consumed) associated with a low reproduction rate but high biomass production. This process is therefore considered as a “cooperative” behavior because the external resources are slowly consumed and are shared with other consumers that colonize the same environment. In contrast, fermentation is a “selfish” behavior characterized by a high rate of glucose consumption and low biomass production due to the use of carbon to produce ethanol (Pfeiffer et al., 2001; MacLean and Gudelj, 2006). In this context, it is interesting to note that in certain LAB species, such as *Lactococcus lactis* and *Lactobacillus plantarum*, heme (and menaquinone) can stimulate aerobic respiration (**Figure 1**), thereby increasing not only growth efficiency but also robustness (Brooijmans et al., 2009). In all of these examples, the shift in metabolic efficiency originates from a tradeoff between investments in enzyme synthesis and metabolic yields from alternative catabolic pathways (Molenaar et al., 2009).

From an ecological point of view, cellular decision-making is also based on the economics of nutrition and competition, and any organism has to find the most efficient way of colonizing its niche (Zakrzewska et al., 2011). In this context, growth rate and robustness are related properties that show a remarkable inverse correlation. Therefore, the net results of the evolutionary selection for microorganisms in constantly changing nutrient-abundant and stress-free environments automatically lead to selection for individuals with reduced robustness and rapid growth (Zakrzewska et al., 2011). More generally, the rate-yield tradeoff of metabolic pathways was experimentally shown to be an evolutionary constraint that reflects the evolutionary history of populations (MacLean, 2008). This tradeoff generates a fundamental social conflict (“the tragedy of the commons”) in microbial populations because the average fitness in a population is highest if all individuals exploit resources efficiently, but an individual’s reproductive rate is maximized by consuming common resources at the highest possible rate (MacLean and Gudelj, 2006; MacLean, 2008). This social and evolutionary dilemma arises only if there is competition for shared resources. Therefore, pathways with a high rate but low yield of ATP production should primarily be observed in association with the exploitation of external resources (Pfeiffer et al., 2001).

It can be assumed that microorganisms use energetically efficient pathways when growing at low concentrations of sugars and energy-wasting pathways at high sugar concentrations, as observed in *S. cerevisiae* (see Crabtree effect), *Bacillus subtilis* (Sonenshein, 2007), and LAB species, which have the ability to shift between low-efficiency homolactic fermentation and high-efficiency mixed acid fermentation (Teusink et al., 2006). In specific, if growing in a medium that contains an excess of glucose *B. subtilis* metabolizes a large proportion of the glucose only as far as pyruvate and acetyl CoA, and subsequently converts these compounds by-products including lactate, acetate, and acetoin (Sonenshein, 2007). For *L. lactis*, product formation at high dilution rates during continuous cultivations is similar to product formation during batch growth at high glucose concentrations, resulting in lactic acid as the sole metabolic product. On the other hand, growth at low dilution rates in continuous conditions or at low concentrations of glucose in batch conditions results in a mixed-acid fermentation (Thomas et al., 1979). On the other hand, *L. plantarum* that is strongly adapted to nutrient-rich environments, even at energy limitations uses a catabolic route that is less efficient in ATP production and uses futile cycles and parallel pathways to uncouple ATP production and anabolic ATP consumption especially under energy excess (Teusink et al., 2006).

The possibility of regulation of the ATP yield and the stoichiometry of oxidative phosphorylation may gain a selective ecological advantage during competition for energy resources. Among the yeasts belonging to the genus *Saccharomyces*, those yeasts that underwent whole-genome duplication exhibit a strong fermentative lifestyle due to the Crabtree effect and the ability to grow under strictly anaerobic conditions. However, only in *S. cerevisiae* have these traits been combined and developed to perfection (Merico et al., 2007). The “make-accumulate-consume ethanol” strategy not only acts to eliminate competitors but also becomes a newly available carbon source for the survivors when glucose is exhausted.

The preferred fermentative mode of energy production are also remarkably evident at the transcriptional regulation level. In this context, it was shown that single transcription-factor deletions can essentially abolish the tricarboxylic acid cycle flux and thus respiration, whereas only the orchestrated action of several glucose signaling pathways shift metabolism toward respiration, thereby inhibiting fermentation (Fendt et al., 2010).

## DID HUMAN SELECTION BASED ON FOOD PROCESSING DRIVE “SELFISH” STRATEGY IN LAB AND YEAST METABOLIC BEHAVIORS?

A fundamental goal in evolutionary biology is to achieve an in-depth understanding of how human activities favor selection for specific metabolic traits in microbial species, guiding these species’ “domestication” or evolution toward new taxa. The question arising here is which type of energetic strategy, whether “selfish” or “cooperative” (**Figure 1**) has also been selected during the domestication of microbial species in environments showing predictable fluctuations, such as food niches. If we consider the energetic strategies adopted by a domesticated strain of *S. cerevisiae* and by the LAB species *Streptococcus thermophilus* and *Lactobacillus delbrueckii* subsp. *bulgaricus*, we can speculate that a “selfish” strategy has been evolutionarily selected in order to adapt to grape juice and milk environments, respectively. Thus, the role of human activities related to food forced the domestication of wild strains toward the selection of an energetic tradeoff between rate and yield on behalf of the rate of ATP production per unit of time. In contrast, the absence of close adaptation to a defined environmental niche should lead to an oscillation between selfish and cooperative behaviors that depends on environmental stimuli. In dairy LAB, the selective forces shaping microbial genotypes and phenotypes in *L. lactis* during evolutionary adaptation from the plant to the milk have been corroborated at the kinetic level based on the differences observed between plant and dairy *L. lactis* lactate dehydrogenase regulation. Dairy strains use fructose-1,6-biphosphate and P_i_, whereas the lactate dehydrogenase of plant isolates is regulated by the NADH/NAD^+^ ratio (Levering et al., 2012). As a plant environmental niche is relatively devoid of phosphate, these observations underline a strict link between the organism’s natural environment and its metabolic regulation. More recently (Bachmann et al., 2012), in a plant-derived *L. lactis* strain, adaptation to a new environment, milk, was achieved in only the 1000th generation and resulted in an increased growth rate, yield, and fitness, together with a loss of mobile elements. More interestingly, it was observed that the transcriptome of milk-adapted strains converged toward the transcriptome of a dairy environment-derived reference strain (Bachmann et al., 2012), indicating an extremely rapid evolution of the first layer of regulatory mechanisms in response to the appearance of new environmental constraints. In particular, the new milk-adapted strains were characterized by the downregulation of transport systems dedicated to plant sugars and branched-chain amino acid biosynthesis, thus reflecting the ongoing process of genomic decay during adaptation to the milk environment. This genomic decay or regressive evolution linked to milk adaptation was already described as a key feature of the dairy species *S. thermophilus* and *L. delbrueckii* subsp. *bulgaricus*, the two species involved in yogurt production (Bolotin et al., 2004; van de Guchte et al., 2006). Genomic analysis of the latter species indicated its adaptation from a plant-associated habitat to the stable protein- and lactose-rich milk environment through the loss of superfluous functions and protocooperation with *S. thermophilus* (van de Guchte et al., 2006). Both the *S. thermophilus* and *L. delbrueckii* species are characterized by homofermentative lactic acid metabolism and do not have any heme-dependent respiratory metabolism, as observed for other LAB that are “still” able to colonize different ecological niches, as demonstrated by their metabolic and genetic flexibility (Brooijmans et al., 2009). It can therefore be hypothesized that adaptation to a dairy environment was generated by human activities that selected for microorganisms with “selfish metabolic behavior. Moreover, the phenotypic characteristics of *S. salivarius*, the closed phylogenetic neighbor of *S. thermophilus*, are further evidences of adaptation to milk environment by the latter species. *S. salivarius* is inhabitant of the oral cavity of mammals and despite the high phylogenetic relationship with *S. thermophilus*, these two species show an extremely different carbohydrate utilization pattern (Schleifer et al., 1991; Mora et al., 2002) and are characterized by differences in the transcriptional regulation of urease operon (Chen et al., 1998; Mora et al., 2005) that reflects the fluctuations of nutrients availability in milk and in the oral cavity of mammals.

The description and comparative genomic analysis of the LAB *S. thermophilus* (Bolotin et al., 2004) represented the first report providing insights into adaptive evolutionary mechanisms that led to the assembly of a “generally recognized as safe” (GRAS) species from closely related deadly human pathogens, such as *S. pneumoniae*, *S. pyogenes*, and *S. agalactiae*. Moreover, Bolotin et al. (2004) argued that the species *S. thermophilus* evolved from closely phylogenetically related pathogenic streptococci through loss-of-function events counterbalanced by the acquisition of relevant traits, such as lactose and urea utilization (Bolotin et al., 2004; Arioli et al., 2010), that have allowed the assembly of new genomic organization suitable for the colonization of the dairy niche.

In a more general view we can assume that the adaptation process to food environments it is far to be known because several food fermenting bacteria are clearly not adapted to food fermentation because distinct food-derived lineages do not exist. This is particularly true for *L. plantarum* and *Lactobacillus reuteri* that are involved in cereal fermentations since several millennia (Hayden et al., 2013). For *L. plantarum* the absence of the adaptation to a unique food-niche it is witnessed by an extremely high genome plasticity where the main features appears to be genomic life-style islands consisting of numerous functional gene cassettes, in particular for carbohydrates utilization, which can be acquired, shuffled, substituted, or deleted in response to niche requirements (Siezen and van Hylckama Vlieg, 2011). *L. reuteri* is both a gut symbiont and a stable member of sourdough microbiota (Walter et al., 2011; Su et al., 2012). Interestingly, it was recently demonstrated that sourdough isolates of *L. reuteri* emerge from the same phylogenetic line as rodent strains (Su et al., 2012) and it was excluded that the stable microbiota of sourdough could be subjected by recurrent fecal contamination of raw material. Additionally, it was reported that rodent *L. reuteri* isolates are capable of long-term persistence in food fermentation and that sourdough isolates were able to colonize *Lactobacillus*-free mouse model (Walter et al., 2008). It therefore appeared that *L. reuteri* can stably colonize two environmental niches, the upper intestine of mammals that consume cereal-based foods and sourdough, due to the similarities between the two habitats, i.e., availability of sucrose and maltose as the major carbon source (Su et al., 2012).

Has the domestication of *S. cerevisiae* caused similar metabolic selection? *S. cerevisiae* has been used for millennia in baking, brewing, and winemaking, and more recently, for ethanol production as a biofuel. All of these biotechnological applications are based on the very efficient fermentative metabolism of the yeast. Piškur et al. (2006) speculated that efficient metabolic regulation was the most unique “invention” of *Saccharomyces* yeast used in the industrial fermentations of breweries and wineries. The ability of *S. cerevisiae* to use respiro-fermentative metabolism has been an important characteristic in the evolutionary and ecological contexts and in many of the yeast’s industrial applications.

Progress in genomic sequencing has elucidated the phylogenetic relationships among yeasts and has revealed the importance of genomic events, such as whole-genome duplication (Wolfe and Shields, 1997), in the evolutionary history of the yeast, which occurred in the *S. cerevisiae* lineage approximately 100 mya, and the rewiring of the expression of hundreds of genes (Ihmels et al., 2005). Whole-genome duplication in yeast determined an increased glycolytic flux and therefore an increased fermentative capacity, which is a great advantage when glucose resources are both large and dense (Conant and Wolfe, 2007). Nevertheless, population genetic studies showed that *S. cerevisiae* strains associated with human activities are differentiated from the natural wild populations (Fay and Benavides, 2005; Liti et al., 2009). The recent analysis of wild *S. cerevisiae* population collected from primeval and rainforests across China (Wang et al., 2012) is now opening a new “path” to expand the knowledge about yeast’s history. The study of genetic, as well as of metabolic traits of these new and wild yeast lineages, could in fact keep many surprises and furthermore help to elucidate yeast genetic and metabolic evolution. It is noteworthy that so far most of what we know about *S. cerevisiae* is mainly based on studies carried out on laboratory strains.

Likewise on yeast, also in LAB genomic events resulted in new metabolic traits. A high relative activity of several glycolytic enzymes per cell, an outcome of diploidy, was measured in dairy strains of *L. lactis* (Michelsen et al., 2010). Although bacteria are normally haploid, maintaining one copy of their genome, several dairy strains of *L. lactis* fulfill the criterion for diploidy. The diploid dairy strains show higher sensitivity to UV light, increased cell size and higher glycolytic activity compared with haploid non-dairy strains. The authors concluded their study by proposing that diploidy has been selected during the 5,000–10,000 years that lactococci have been used in cheese production (Michelsen et al., 2010). Whole-genome duplication in yeast and diploidy in *L. lactis* were most likely events in a long process of adaptation that led to modern baker’s yeast and dairy lactococci, under the selective pressure of human food processing.

## CONCLUSION

Food matrixes represent natural environments colonized by microorganisms, and fermentation is one of the oldest forms of food preparation and preservation. Here, we discussed how these environments have shaped the primary energetic metabolism of food-associated microorganisms, using the yeast *S. cerevisiae* and species of LAB known to be strictly adapted or “domesticated” to a defined food niche as examples. These microorganisms are characterized by a high phylogenetic distance but share similar cellular bioenergetics due to their evolution in food environments rich in easily assimilable carbon and nitrogen sources.

The adaptation to these environmental contexts caused genomic and metabolic streamlining in certain cases, resulting in a “reductive evolution” in which simple organisms derive from more complex ancestors (Bolotin et al., 2004; van de Guchte et al., 2006). Moreover, in such microorganisms, adaptation to food matrixes selected for “selfish” energetic behavior and for bioenergetic regulatory mechanisms that remarkably mirror the predictable environmental changes occurring during growth (i.e., during the fermentation process; Mitchell et al., 2009). The ecological forces and molecular mechanisms that govern this ability are not clear, but it is evident that the regulatory networks that link environmental stimuli to microbial responses are complex and can evolve rapidly. In conclusion, the bioenergetics of food-associated bacteria should be analyzed, always considering the environmental context, nutrient richness and availability, and the “predictable” succession of environmental stimuli that have driven the organisms’ domesticated speciation and evolution.

## Conflict of Interest Statement

The authors declare that the research was conducted in the absence of any commercial or financial relationships that could be construed as a potential conflict of interest.
